# Resection of NAFLD/NASH-related Hepatocellular Carcinoma (HCC): Clinical Features and Outcomes Compared with HCC Due to Other Etiologies

**DOI:** 10.1093/oncolo/oyac251

**Published:** 2023-02-10

**Authors:** Surendra Pal Chaudhary, Stephanie Reyes, Matthew L Chase, Aparna Govindan, Lei Zhao, Jay Luther, Irun Bhan, Emily Bethea, Joseph W Franses, Elizabeth Paige Walsh, Leigh Anne Dageford, Shoko Kimura, Nahel Elias, Heidi Yeh, James Markman, Adel Bozorgzadeh, Kenneth Tanabe, Cristina Ferrone, Andrew X Zhu, Karin Andersson, Michael Thiim, Onofrio Antonio Catalano, Avinash Kambadakone, Parsia A Vagefi, Motaz Qadan, Daniel Pratt, Nikroo Hashemi, Kathleen E Corey, Joseph Misdraji, Lipika Goyal, Jeffrey W Clark

**Affiliations:** Division of Oncology, Mass General Cancer Center and Harvard Medical School, Boston, MA, USA; Duke University School of Medicine, Durham, NC, USA; Beth Israel Deaconess Hospital, Needham, MA, USA; Icahn School of Medicine at Mount Sinai, New York, NY, USA; Department of Pathology, Brigham and Women’s Hospital, Harvard Medical School, Boston, MA, USA; Division of Gastroenterology, Massachusetts General Hospital and Harvard Medical School, Boston, MA, USA; Division of Gastroenterology, Massachusetts General Hospital and Harvard Medical School, Boston, MA, USA; Division of Gastroenterology, Massachusetts General Hospital and Harvard Medical School, Boston, MA, USA; Division of Oncology, Mass General Cancer Center and Harvard Medical School, Boston, MA, USA; Division of Oncology, Mass General Cancer Center and Harvard Medical School, Boston, MA, USA; Transplantation Unit, Department of Surgery, Massachusetts General Hospital and Harvard Medical School, Boston, MA, USA; Transplantation Unit, Department of Surgery, Massachusetts General Hospital and Harvard Medical School, Boston, MA, USA; Transplantation Unit, Department of Surgery, Massachusetts General Hospital and Harvard Medical School, Boston, MA, USA; Transplantation Unit, Department of Surgery, Massachusetts General Hospital and Harvard Medical School, Boston, MA, USA; Transplantation Unit, Department of Surgery, Massachusetts General Hospital and Harvard Medical School, Boston, MA, USA; Transplantation Unit, Department of Surgery, Massachusetts General Hospital and Harvard Medical School, Boston, MA, USA; Department of Surgery, Massachusetts General Hospital and Harvard Medical School, Boston, MA, USA; Department of Surgery, Massachusetts General Hospital and Harvard Medical School, Boston, MA, USA; Jiahui Health, Jiahui International Cancer Center, Shanghai, People’s Republic of China; Division of Gastroenterology, Massachusetts General Hospital and Harvard Medical School, Boston, MA, USA; Division of Gastroenterology, Massachusetts General Hospital and Harvard Medical School, Boston, MA, USA; Department of Radiology, Massachusetts General Hospital and Harvard Medical School, Boston, MA, USA; Department of Radiology, Massachusetts General Hospital and Harvard Medical School, Boston, MA, USA; Division of Surgical Transplantation, University of Texas Southwestern, Dallas, TX, USA; Department of Surgery, Massachusetts General Hospital and Harvard Medical School, Boston, MA, USA; Division of Gastroenterology, Massachusetts General Hospital and Harvard Medical School, Boston, MA, USA; Division of Gastroenterology, Hepatology and Endoscopy, Brigham and Women’s Hospital and Harvard Medical School, Boston, MA, USA; Division of Gastroenterology, Massachusetts General Hospital and Harvard Medical School, Boston, MA, USA; Department of Pathology, Yale New Haven Hospital, Yale University, New Haven, CT, USA; Division of Oncology, Mass General Cancer Center and Harvard Medical School, Boston, MA, USA; Division of Oncology, Mass General Cancer Center and Harvard Medical School, Boston, MA, USA

**Keywords:** steatohepatitis, NAFLD, NASH, hepatocellular carcinoma, outcome, metabolic syndrome

## Abstract

**Background:**

Non-alcoholic fatty liver disease (NAFLD) and non-alcoholic steatohepatitis (NASH) are the leading causes of hepatocellular carcinoma (HCC) worldwide. Limited data exist on surgical outcomes for NAFLD/NASH-related HCC compared with other HCC etiologies. We evaluated differences in clinicopathological characteristics and outcomes of patients undergoing surgical resection for NAFLD/NASH-associated HCC compared with other HCC etiologies.

**Methods:**

Demographic, clinicopathological features, and survival outcomes of patients with surgically resected HCC were collected. NAFLD activity score (NAS) and fibrosis score were assessed by focused pathologic review in a subset of patients.

**Results:**

Among 492 patients screened, 260 met eligibility (NAFLD/NASH [*n* = 110], and other etiologies [*n* = 150]). Median age at diagnosis was higher in the NAFLD/NASH HCC cohort compared with the other etiologies cohort (66.7 vs. 63.4 years, respectively, *P* = .005), with an increased percentage of female patients (36% vs. 18%, *P* = .001). NAFLD/NASH-related tumors were more commonly >5 cm (66.0% vs. 45%, *P* = .001). There were no significant differences in rates of lymphovascular or perineural invasion, histologic grade, or serum AFP levels. The NAFLD/NASH cohort had lower rates of background liver fibrosis, lower AST and ALT levels, and higher platelet counts (*P* < .01 for all). Median overall survival (OS) was numerically shorter in NAFLD/NASH vs other etiology groups, however, not statistically significant.

**Conclusions:**

Patients with NAFLD/NASH-related HCC more commonly lacked liver fibrosis and presented with larger HCCs compared with patients with HCC from other etiologies. No differences were seen in rates of other high-risk features or survival. With the caveat of sample size and retrospective analysis, this supports a similar decision-making approach regarding surgical resection for NAFLD/NASH and other etiology-related HCCs.

## Teaser

This article evaluates differences in clinicopathological characteristics and outcomes of patients undergoing surgical resection for non-alcoholic fatty liver disease-associated and non-alcoholic steatohepatitis-associated hepatocellular carcinoma compared with other etiologies.

**Implications for Practice**

There is an increasing prevalence of NAFLD/NASH-related HCC, especially in the Western world. The complexity of disease biology and the absence of effective screening guidelines, approved biomarkers, or effective treatment for NAFLD/NASH, makes it imperative to understand the key aspects of this disease better. Our study findings contribute to an enhanced understanding of the disease biology and outcome of NAFLD/NASH-related HCC in patients who underwent surgical resection. The fact that NAFLD/NASH patients were diagnosed with larger tumors highlights the importance of developing biomarkers to predict which patients with NAFLD and NASH will develop HCC to aid in the ability to better screen appropriate patients for the development of HCC.

## Introduction

Hepatocellular carcinoma (HCC) accounts for approximately 80% of primary liver cancers and is the fourth leading cause of cancer-related deaths worldwide.^1^ HCC most commonly occurs in the setting of chronic inflammation and cirrhosis although it can occur in the absence of cirrhosis. Risk factors for HCC include infections (chronic viral hepatitis B [HBV] and hepatitis C [HCV]), excessive alcohol intake, metabolic syndrome (MS)-associated non-alcoholic fatty liver disease (NAFLD), and non-alcoholic steatohepatitis (NASH), exposure to environmental toxins such as aflatoxin, inherited genetic diseases such as hemochromatosis or alpha1-antitrypsin deficiency, autoimmune hepatitis, and primary biliary sclerosis.^2,3^ Treatment options for HCC vary by the stage of the disease. For early-stage HCC, the treatments of choice include surgical resection, ablation, and orthotopic liver transplant (OLT).^4,5^

Metabolic syndrome (MS) is a clinical diagnosis defined by the presence of 3 of the following 5 conditions: abdominal obesity, elevated triglycerides, reduced high-density lipoprotein (HDL), hypertension, and impaired fasting glucose.^6^ It is the major risk factor for the development of NAFLD which is characterized by hepatic steatosis by histology or imaging in the absence of a history of significant alcohol consumption or other known liver diseases. Non-alcoholic steatohepatitis (NASH), a progressive form of NAFLD, currently can only be accurately diagnosed histologically and is characterized by ballooning hepatocellular injury often accompanied by intracytoplasmic aggregated cytokeratin intermediate microfilaments (Mallory–Denk bodies) and lobular inflammation.^7^ Patients with NASH have an increased risk for the development of liver fibrosis, cirrhosis, and HCC.^6^

Currently, NAFLD is estimated to be the most common cause of chronic liver disease in the United States, affecting approximately 24% of adults and up to 10% of children, with approximately 10%-25% of those with NAFLD progressing to NASH over time.^8,9^ A meta-analysis of 9 studies that included greater than 1.5 million participants reported that obesity, defined as a body mass index (BMI) greater than 30 kg/m^2^, was associated with a 2-fold increased risk of developing HCC.^10,11^ Recent studies have also shown that lean individuals, especially in Asia, can develop NAFLD^11^ although the risk of HCC in patients with “lean NAFLD” is not as well defined. An estimated 20%-30% of patients with NASH will develop fibrosis, with 3%-15% developing cirrhosis. The annual incidence rate of HCC in those with NASH is estimated at 5.29 per 1000 person-years.^12^ The global prevalence of NAFLD and NASH continues to rise, and they are emerging as an increasingly common etiology for HCC in both developed and developing countries.^12,13^

Previous studies have sought to identify differences in patient demographics and co-morbidities between patients with NAFLD/NASH HCC and non-NASH HCC, as well as different pathways of tumorigenesis.^12,14–18^ Studies evaluating survival outcomes of patients with HCC arising in NASH versus other etiologies (primarily viral and/or alcohol-related) treated with definitive local therapy (various combinations of transplant, surgery, and ablation) have reached mixed conclusions. While most studies report equivalent relapse-free and overall survival (OS), some studies have demonstrated worse OS with NASH-related HCC compared with combined cohorts of other etiologies, worse OS compared with alcohol-related liver disease (ALD)-associated HCC, and improved survival compared with viral hepatitis-associated HCC.^14–18^ Few studies have reported specifically on the subset of patients who have had a surgical resection for HCC. Therefore, to address potential differences in this population, we compared patient demographics, co-morbidities, disease characteristics, and clinical outcomes between patients with surgically resected HCC due to NAFLD/NASH and or other etiologies in a large multi-institutional study.

## Patients and Methods

In this retrospective study, pathology databases were used to identify patients who underwent surgery for HCC between February 2004 and April 2015 at the Massachusetts General Hospital (MGH) or Brigham and Women’s Hospital, and study follow-up was completed in 2019. The terms “HCC”, “hepatectomy”, “hepatoma,” and “hepatic resection” were used as search terms. The NAFLD/NASH cohort included patients with a NAFLD activity score (NAS) of ≥1^6^ as scored by an attending pathologist (J.M.) or met 3 of 5 criteria for MS defined by the National Cholesterol Education Program’s Adult Treatment Panel III report (Table 1).^19^ Patients with cryptogenic cirrhosis were included in the NAFLD/NASH cohort, and patients with a history of significant alcohol consumption or other chronic liver diseases were excluded. The cohort of patients with other chronic liver diseases (the “other etiologies cohort”) included those with a known history of chronic HCV, chronic HBV, alcohol abuse, hemochromatosis, or other etiology. Patients were confirmed as having chronic HBV or HCV by serology (HBV surface antigen or DNA positivity; HCV antibody or RNA positivity), and/or they were receiving or had received antiviral therapy for either. Patient medical records were used to determine alcohol consumption. For the patients who received multiple curative intent surgeries for HCC, only the first operation was included in the study. Data were collected retrospectively from the electronic medical record system on a protocol approved by the Partners Institutional Review Board.

**Table 1.** Cohort’s characteristics.

**Cohort #1—NAFLD/NASH** **+** **cryptogenic cohort (metabolic risk factor related HCC) is defined as:**
No history of chronic HCV, HBV, alcohol abuse, hemochromatosis, autoimmune hepatitis, or primary sclerosing cholangitis
And
• Three of the following five characteristics for the adult treatment plan (ATPIII) definition of MS
◦ BMI >28.8
◦ On an anti-hypertensive medication or BP ≥130/≥85 mmHg on two occasions
◦ On a diabetes medication or HbA1c >6.5%
◦ On a triglyceride-lowering agent or triglycerides ≥150 mg/dL
◦ On a lipid-lowering agent or an HDL for men <40 mg/dL, for women <50 mg/dL
Or
• NASH activity score (NAS) of ≥1 with at least one point for steatosis
◦ Steatosis (0–3)
◦ Lobular inflammation (0–3)
◦ Hepatocellular ballooning (0–2)
◦ Brunt fibrosis (0–4)
Cryptogenic: Cryptogenic cause cirrhosis (CC) is defined as the end stage of a chronic liver disease in which its underlying etiology remains unknown after extensive clinical, serological, and pathological evaluation
**Cohort #2—Non-NASH or other etiologies cohort is defined as**
• Known history of chronic HCV, chronic HBV, alcohol abuse (in notes), hemochromatosis, or other etiologies besides NASH

Abbreviations: ATPIII, adult treatment panel; BMI, body mass index; HBV, chronic viral hepatitis B; HCV, chronic viral hepatitis C; HDL, high-density lipoprotein; MS, metabolic syndrome; NASH, non-alcoholic steatohepatitis; NAS, NASH activity score.

### Rationale for Inclusion of HCC Patients Arising in the Background of Cryptogenic Cirrhosis with the NAFLD/NASH Cohort

Although there are multiple potential etiologies for cryptogenic HCC, the majority of these are thought to be due to NAFLD that was not previously recognized.^20^ This is due to several factors. Not all of the features of MS need to be present for NAFLD and NASH to develop. The absence of central obesity is not a reliable factor for ruling out NASH as up to 25% of patients with NASH do not have central obesity, especially in some Asian countries.^21^ In addition, there are histologic features that suggest many cases of cryptogenic cirrhosis likely had a NASH etiology.^22^ Thus, especially before the past decade when NASH became recognized as a common etiology for HCC, a high percentage of these cases would likely have been interpreted as cryptogenic. Tumor size was based on explant pathology, not pre-surgical imaging, and was defined as the largest tumor diameter. For multiple tumors, the sum of the largest tumor diameter of each lesion was used.

## Data Collection and Statistical Plan

The data analyzed included demographic factors, clinical risk factors, laboratory values, tumor pathologic features, and survival outcomes. Scoring fibrosis of the background liver was based upon the Brunt and Ishak scoring system,^6,23^ depending on the etiology of liver disease. Brunt scoring was scaled 0-4 and Ishak scoring was scaled 0-6. For fibrosis, scores of 0-1 were labeled as “absent” and ≥2 as “present”. In cases where fibrosis was not reported in the pathology report, an attending pathologist (J.M.) staged the cases with available tissue based on trichrome stains prepared at the time of initial evaluation.

Continuous variables were compared using the Student *t*-test. Categorical variables were compared using the chi-square test. Median recurrence-free survival (RFS) and OS after liver resection were calculated using the Kaplan-Meier method and compared using the log-rank test. Categorical and time-to-event outcomes were compared using the chi-square test and Cox proportion hazard regression. Assumptions of baseline hazard proportionality were assessed for each of the available covariates and predictors. In the first stage, a univariate Cox regression model was used to identify the eligible predictors of mortality which showed a marginal association of 10% with the primary outcome (alive vs. deceased). In the second stage, a backward stepwise multiple Cox proportional hazard model was used to identify the independent predictors of mortality with an entry *P* = .1 and exit *P* = .05. All analyses were performed using STATA 15.1 (StataCorp, College Station, TX) and 2-sided nominal *P* < .05 were considered for statistical significance.

## Results

**Figure 1.**
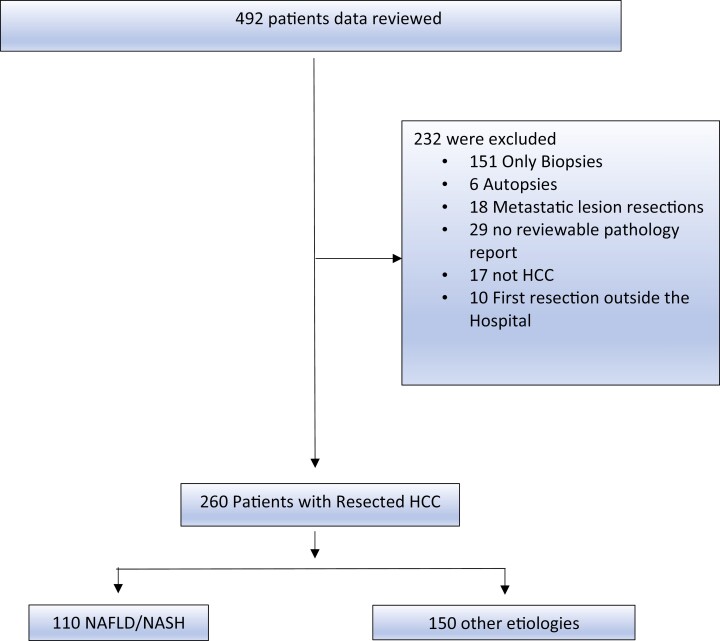
CONSORT Diagram of the study depicting screened and eligible populations, reasons for exclusion, and break down of patients into the NAFLD/NASH and other etiologies cohorts.

Of the 492 patients identified for this study, 260 patients met the eligibility criteria. The most common reason for exclusion included biopsy specimens only with no history of resection (*n* = 152) (Fig. 1). Of 260 patients with resected HCC, the presumed cause of HCC was NAFLD/NASH in 61 cases, cryptogenic in 49, and other defined etiology in 150. A subset of patients with a cryptogenic HCC were grouped into the NAFLD/NASH cohort as explained in the Patients and Methods section. The other etiologies cohort included patients with chronic HBV (27%, *n* = 41), chronic HCV (36%, *n* = 54), concurrent chronic hepatitis B and C (4%, *n* = 6), alcoholic-related cirrhosis (29%, *n* = 43), hemochromatosis (3.3%, *n* = 5), and alpha-1 antitrypsin deficiency (0.67%, *n* = 1). Baseline characteristics for the resection group are shown in Table 2.

**Table 2.** Baseline characteristics of patients with HCC in the NAFLD/NASH and other etiologies cohorts.

Variables	NAFLD/NASH *N* = 110 (42%)	Other etiologies *N* = 150 (58%)	*P*-value
Gender, *n* (%)			.001
Male	70 (63.3)	123 (82.0)	
Female	40 (36.0)	27 (18.0)	
Median age at surgery (years)	66.7	63.4	.005
Race, *n* (%)			.001
Caucasian	69 (62.7)	70 (46.6)	
Black	0 (0)	9 (6.0)	
Hispanic	4(3.6)	2 (1.3)	
Asian	4 (3.6)	34 (22.0)	
Other	1 (0.9)	0 (0)	
Unknown	32 (29.0)	35 (23.0)	
Risk factors, *n* (%)			
HBV	0 (0)	41 (27.3)	
HCV	0 (0)	54 (36.0)	
HBV + HCV	0 (0)	6 (4)	
EtOH	0 (0)	43 (28.7)	
Hemochromatosis	0 (0)	5 (3.3)	
Other (alfa-1 antitrypsin deficiency)	0 (0)	1 (0.67)	
Cryptogenic	49 (19)	0 (0)	
MS	60 (55.0)	36 (24.0)	.001
Diabetes	51 (50.0)	41 (25.0)	.001
Hypertension	84 (76.0)	98 (65.3)	.037
Hypercholesterolemia	57 (51.8)	44 (29.3)	.001
Pre-surgical BMI ≥ 28.8	46 (43.4)	37 (25.0)	.003
Median pre-surgical labs			
Total bilirubin	0.5	0.6	.14
Albumin	4.0	4.2	.06
Platelets	252	175	.0001
INR	1.1	1.1	.58
ALT	34	45	.004
AST	36	50	.003
AFP	10.1	10.6	.23
Cr	0.98	0.94	.29
MELD	8.1	7.4	.21
Ascites, *n* (%)	7 (6.3)	8 (5.0)	.72
Hepatic encephalopathy, *n* (%)	0 (0)	3 (2.0)	.13

Abbreviations: BMI, body mass index; HBV, chronic viral hepatitis B; HCC, hepatocellular carcinoma; HCV, chronic viral hepatitis C; MS, metabolic syndrome; NAFLD, non-alcoholic fatty liver disease; NASH, non-alcoholic steatohepatitis.

### Comparison of Patient Demographics and Co-morbidities in NAFLD/NASH and Other Etiologies Cohorts

The median age at diagnosis was higher in the NAFLD/NASH cohort compared with the other etiologies cohort (66.7 vs. 63.4 years, respectively, *P* = .005), as was the percentage of female patients (36% vs. 18%, *P* = .001). The NAFLD/NASH population had a higher percentage of White patients compared with other etiologies (63% vs. 47%; *P* = .001), which had a higher percentage of Asians (22% vs. 4%; *P* = .001), the latter due to the increased percentage of cases with chronic HBV. Compared with the cohort of patients with other etiologies HCC, the cohort of NAFLD/NASH HCC had a significantly higher percentage of patients with BMI >30.0 kg/m^2^ (43% vs. 25.0%, *P* = .003), diabetes mellitus (DM) (83.6% vs. 21.0%, *P* = .001), hypertension (76% vs. 65.0%, *P* = .037), and hypercholesterolemia (52% vs. 29%, *P* = .001).

### Comparison of Background Liver on Pathology and of Surrogates of Liver Function on the Laboratory Analysis

Liver fibrosis scoring was available for 102 (39.0%) patients by pathology reports and performed on 260 available cases by an attending pathologist. This data subset included 78 patients in the NAFLD/NASH cohort and 24 patients in the other etiologies cohort. Liver fibrosis was found to be more commonly present in patients with other etiologies of HCC compared with NAFLD/NASH (70.8% vs. 37.1%, *P* = .005). On baseline laboratory analysis before resection, median ALT and AST were higher in patients with other etiologies compared with NAFLD/NASH (ALT: 45 IU/L vs. 34 IU/L, *P* = .004; AST: 50 IU/L vs. 36 IU/L, *P* = .003) (Table 2). The median platelet count for NAFLD/NASH patients was higher (252 × 10^9^/L vs. 175 × 10^9^/L, *P* < .001). Median MELD scores and the presence of ascites were not significantly different between those with NAFLD/NASH and other etiologies.

### Comparison of Tumor Biology in NAFLD/NASH HCC vs HCC Due to Other Etiologies

The tumor characteristics of the patients with NAFLD/NASH HCC and non-NASH HCC were evaluated (Table 3). Patients with NAFLD/NASH HCC, when compared with those with other etiologies HCC, had a higher median tumor size (6.25 vs. 4.5 cm, *P* = .001) and more frequently had a tumor size ≥5 cm (66% vs. 45%, *P* = .001). However, no differences were seen in rates of vascular invasion, perineural invasion, or in histologic grade. Similarly, no difference was seen in rates of multifocal tumors. Median AFP values were similar in the two groups (NAFLD/NASH, 10 ng/mL; other etiologies, 10.6 ng/mL).

**Table 3.** Pathologic characteristics and staging of patients with HCC in the NAFLD/NASH and other etiologies cohorts.

Variables	NAFLD/NASH *N* = 110 (42%)	Other etiologies *N* = 150 (58%)	*P*-value
Single tumor			.45
No	12 (11.0)	33 (22.0)	
Yes	71 (65.0)	79 (52.6)	
Unknown	27 (24.5)	38 (25.0)	
Tumor size, *n* (%)			.001
<5 cm	37 (33.0)	81 (54.0)	
≥5 cm	73 (66.0)	68 (45.0)	
Median tumor size	6.25	4.5	.001
Vascular invasion			.90
Present	43 (39.0)	55 (36.6)	
Absent	60 (54.5)	86 (57.3.0)	
Unknown	7 (6.3)	9 (6.0)	
Perineural invasion			.79
Present	1 (0.9)	3 (2.0)	
Absent	67 (60.1)	93 (62.0)	
Unknown	42 (38.1)	54 (36.0)	
Biliary invasion			.28
Present	5 (4.5)	2 (1.3)	
Absent	66 (60.0)	96 (64.0)	
Unknown	39 (35.4)	52 (34.6)	
Positive lymph node			.49
Yes	1 (0.91)	0 (0)	
No	17 (15.4)	25 (16.6)	
Unknown	92 (83.6)	125 (83.3)	
Histology, *n* (%)			.71
Well differentiated	18 (16.3)	23 (15.0)	
Well to moderately	7 (6.5)	9 (6.0)	
Moderately differentiated	63 (57.0)	83 (55.3)	
Moderately to poor	10 (9.0)	11 (7.3)	
Poorly differentiated	6 (5.5)	17 (11.3)	
Unknown	6 (5.5)	7 (5.0)	
Liver fibrosis at baseline			.005
Absent	49 (62.8)	7 (29)	
Present	29 (37.1)	17 (70.8)	
Margin status (next to *R*1 < 1 mm)			.46
*R*0	93 (84.5)	123 (82.0)	
*R*1	15 (13.6)	20 (13.3)	
*R*2	2 (1.8)	7 (4.6)	
Stage			.16
I	40 (36.0)	70 (46.6)	
II	47 (42.7)	55 (37.0)	
III	21 (19.0)	25 (16.7)	
IV	2 (1.8)	0 (0)	

Abbreviations: HCC, hepatocellular carcinoma; NAFLD, non-alcoholic fatty liver disease; NASH, non-alcoholic steatohepatitis.

### Survival Analysis for Resected Patients

**Figure 2.**
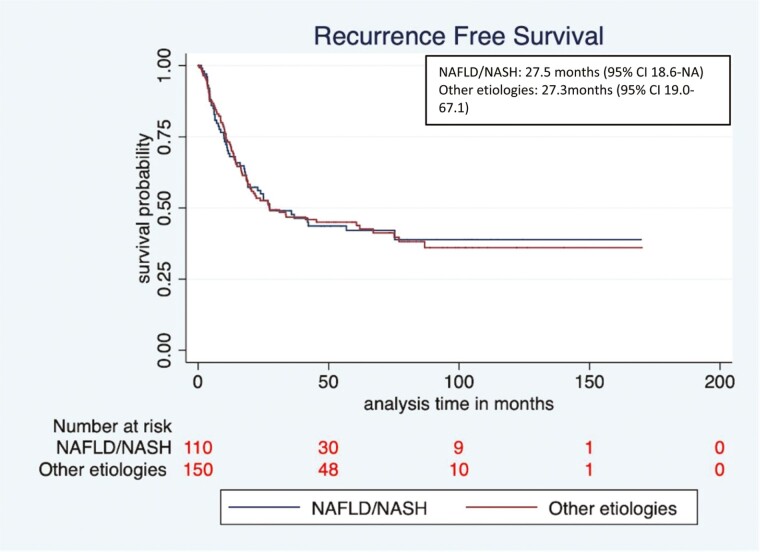
Recurrence-free survival (RFS) of NAFLD/NASH versus other etiologies cohorts.

**Figure 3.**
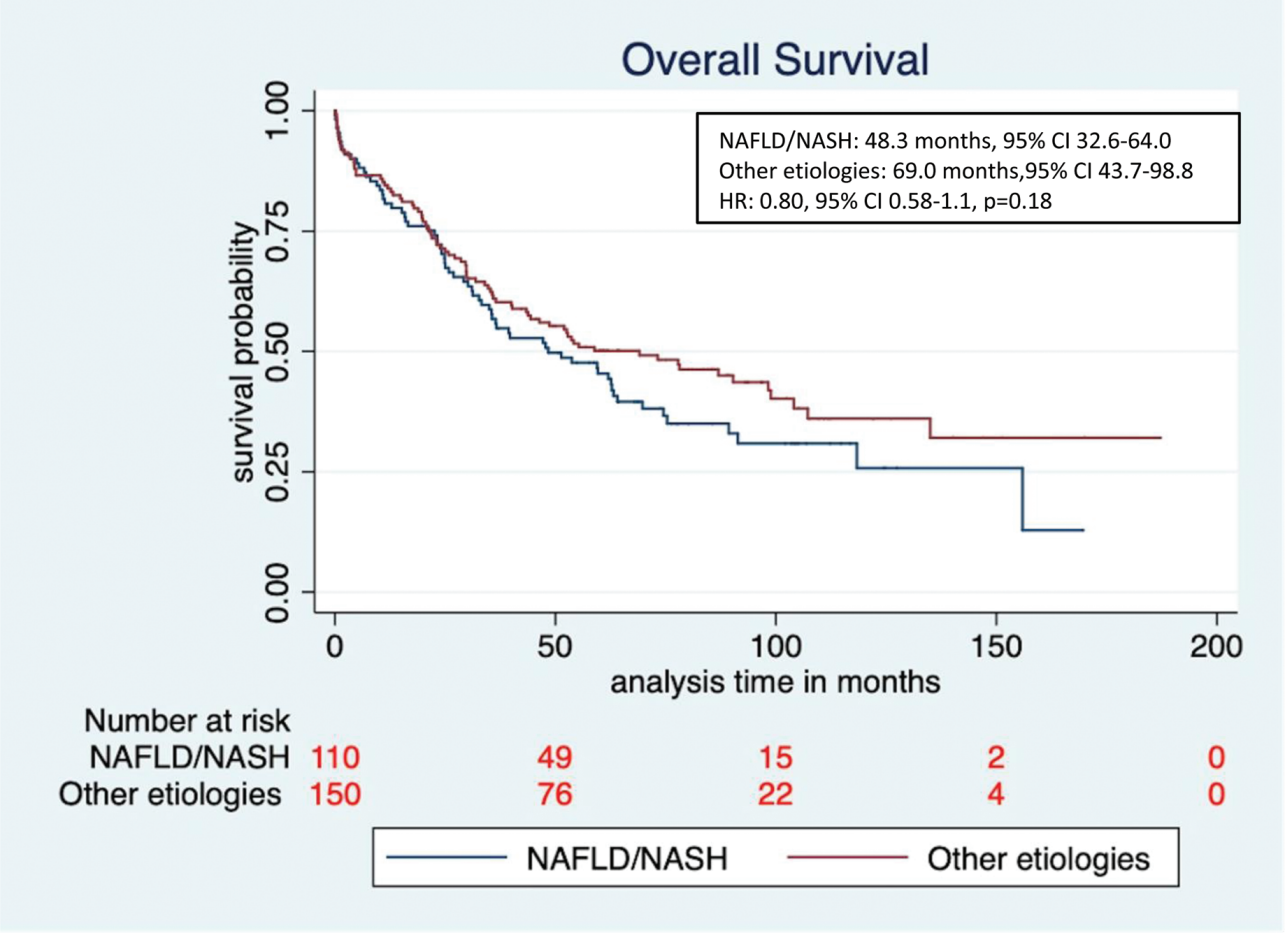
Overall survival (OS) NAFLD/NASH vs other etiologies cohorts.

Recurrence-free survival (RFS) and OS of patients in the NAFLD/NASH and other etiologies cohorts were evaluated. Median RFS was similar among patients with NAFLD/NASH vs other etiologies (27.5 months, 95% CI 18.4–NE vs. 27.3 months, 95% CI 19.0-67.1, respectively; hazard ratio (HR) 1.0, 95% CI 0.7-1.4, *P* = .96). In addition, although the median OS was numerically shorter in the NAFLD/NASH vs. the other etiologies group, this was not statistically significant (48.3 months, 95% CI 33.2-64.0 vs. 69.0 months, 95% CI 43.4-98.8, respectively, HR 0.80, 95% CI 0.58-1.1, *P* = .18) (Figs. 2 and 3). We also evaluated the survival outcomes in patients with NAFLD/NASH versus those with a viral etiology only, and no difference was seen in RFS (27.5 months, 95% CI 18.6-NE vs. 27.2 months, 95%CI 16.5-86.8, respectively; HR 1.0, 95% CI 0.7-1.4, *P* = .90) but there was a trend toward lower median OS in the NAFLD/NASH group (48.3 months, 95% CI 32.6-64.0 vs. 86.9 months, 95% CI 40.1-NE respectively; HR 0.73, 95% CI 0.5-1.0, *P* = .09) (Supplementary Figs. S1 and S2). To be certain that the inclusion of those with cryptogenic cirrhosis combined with those with NAFLD/NASH did not modify the results, we also performed a separate analysis of those with NAFLD/NASH alone versus the combined NAFLD/NASH + cryptogenic patients. No significant difference was observed in the median OS of NAFLD/NASH + cryptogenic versus NAFLD/NASH alone (69.7 months, 95% CI 29.1-NE vs. 47.2 months, 95% CI 32.6-61.9, respectively, HR 1.39, 95% CI 0.85-2.2, *P* = .18). Median OS among those patients who presented with fibrosis at baseline was numerically shorter in the NAFLD/NASH cohort compared with other etiologies cohort but this was not statistically significant (33.2 months, 95% CI 12.8-59.6 vs. 52.2 months, 95% CI 11.9-NE, HR 0.58, 95% CI 0.28-1.22, *P* = .15).

### Univariate and Multivariate Analysis

Univariate and multivariate analysis was performed to determine the independent predictors of survival outcome for the patients who underwent resection of HCC-related to NAFLD/NASH or other etiologies. Factors including age, HBV infection, MS, DM, total bilirubin, AST, platelet count, albumin, INR, serum creatinine, tumor histology, tumor size, tumor involvement of resected margin, and vascular invasion were associated with outcome after liver resection in univariate analysis (*P* < .10) and hence selected for the multivariate analysis (Table 4). The etiology of liver disease was not significantly associated with outcome in the univariate analysis and therefore was not included in the multivariate analysis.

**Table 4.** Stepwise multiple Cox regression model to identify the independent predictors of mortality in resected NAFLD/NASH and other etiologies HCC.

Variable	HR (95% CI)	*P*-value
Age		
<55 (reference)	(reference)	
≥55	1.7 (1.0–2.7)	.033
Diabetes		
No (reference)	(reference)	
Yes	1.7 (1.2–2.5)	.003
Platelet		
Normal (reference)	1 (reference)	
Low	2.6 (1.2–5.4)	.008
Resection		
*R*0 (reference)	(reference)	
*R*1	2.4 (1.5–3.9)	<.001
*R*2	2.7 (1.3–5.7)	.008
Vascular invasion		
No (reference)	(reference)	
Yes	1.5 (1.0–2.0)	.015

Abbreviations: HCC, hepatocellular carcinoma; HR, hazard ratio; NAFLD, non-alcoholic fatty liver disease; NASH, non-alcoholic steatohepatitis.

Multivariate analysis was performed to determine the independent predictors of outcome for the patients who underwent resection. In the multiple Cox regression model, a higher risk of death was seen in patients with age ≥55 years (HR 1.7, 95% CI 1.0-2.7, *P* = .003), diabetes (HR 1.7, 95% CI 1.2-2.5, *P* = .003), and a low platelet count (HR 1.3, 95% CI 1.0-1.7, *P* = .033). As expected, compared with *R*0 resection (microscopically margin-negative resection), patients with *R*1 (microscopic margin are positive for tumor) and *R*2 resections (gross residual tumor and macroscopic margin involvement) had a higher likelihood of mortality (HR 2.4 [95% CI 1.5-3.9], *P* < .001 and HR 2.7 [95% CI 1.3-5.7], *P* = .008, respectively) as did those with vascular invasion compared with those without it (HR 1.5, 95% CI 1.0-2.0, *P* = .015) (Table 4 ).

## Discussion

The rising incidence of NAFLD and NASH globally and the increasing proportion of HCC caused by these underscore the importance of understanding the subset of NAFLD/NASH-related HCC.^24^ In this study comparing clinicopathologic features and prognosis for patients with NAFLD/NASH-related HCC and HCC due to other etiologies, we found that patients with NASH/NAFLD were more commonly female, diagnosed with HCC at an older age, had a higher BMI, had preserved liver function, and a decreased incidence of cirrhosis, similar to what has been reported in previous studies.^17,25^ This study focused specifically on patients who underwent surgical resection for HCC and found that the NAFLD/NASH patients also had a greater median tumor size but did not have higher rates of other pathological features that portend a poor prognosis. The composite effect of liver factors, tumor factors, and patient factors evaluated in this study summed to no significant difference in RFS or OS between the 2 cohorts.

While the larger tumor size seen in patients with NAFLD/NASH did not translate to worse patient outcomes in our study, a trend toward worse survival was seen. Previous studies have shown variable results in differences between patients with NAFLD/NASH and other etiologies, with some studies showing comparable outcomes to other etiologies similar to our results, with others showing worse outcomes than those with alcoholic liver disease but comparable survival to viral hepatitis.^17,26^ These inconsistent findings on the prognosis of NAFLD/NASH-related HCC may be explained by differences in sample size, treatment modalities included, definitions of NAFLD/NASH, and reliability of data capture, all resulting in differences in statistical power to detect distinctions.^14–18,27–30^ Importantly, other studies have included patients treated with a variety of approaches whereas our study evaluated only patients who underwent surgical resection and this may also contribute to differences from some previous studies.

Non-alcoholic fatty liver disease (NAFLD)/NASH-associated HCC is often diagnosed late, possibly at least in part due to decreased screening rates for HCC in this population, and patients with NAFLD/NASH-related HCC continue to have a poor prognosis overall.^12,31^ In addition to surgery, a liver transplant is the other primary potentially curative approach for localized disease. NAFLD/NASH is increasingly an indication for liver transplants in the United States (,^32^ A retrospective study done by the United Network for Organ Sharing and Organ Transplantation (UNOS/OPTN) 2003-2014 database concluded that NASH is the most rapidly growing and second leading indication (after alcohol) of liver transplant in the United States.^33^ Shinginia et al reported that NASH, with or without HCC, related liver transplants have increased over time, especially among younger individuals.^34^

Prevention provides the best approach for decreasing the morbidity and mortality associated with NAFLD and associated HCC. Broadly educating people about the importance of a healthy diet and exercise to decrease the incidence of obesity and MS is most important. In addition, better diagnostic and prognostic biomarkers for NAFLD/NASH and HCC are needed to improve outcomes.^35,36^ Multiple factors influence whether and to what extent NAFLD will progress to NASH and HCC, including differences in progression or regression of causes of MS, insulin resistance, other endocrinologic factors such as growth hormone deficiency, fat storage mechanisms, lipid metabolism, presence of lobular and portal inflammation, oxidative and/or endoplasmic reticulum stress, altered immune responses, mitochondrial dysfunction, signaling networks, inflammatory cytokine production, alterations in gut microbiota, complex genetic variation, and epigenetic changes.^36–41^ This complexity, as well as gaps in knowledge regarding the critical factors that drive progression to HCC, has limited biomarker development. While several non-invasive circulating biomarkers or imaging approaches to monitor this NAFLD/NASH progression have been analyzed, none of these have yet been established as standards of care.^42–44^ Additionally, early detection of HCC remains a challenge in the NAFLD/NASH population. The population of patients with NAFLD/NASH is too large globally to cost-effectively screen everyone with current surveillance protocols, and therefore more effective, less invasive, and more accessible clinical, serum, and imaging biomarkers are needed for risk stratification.

Finally, novel therapeutic interventions are needed both to prevent the progression of NAFLD to its serious complications and to decrease the progression or recurrence of HCC. Currently, despite extensive study and drug development, no therapeutic agents have gained FDA approval for reducing the progression of NAFLD to NASH or for the treatment of NASH.^45-49^ Examples of ideas being explored include approaches targeting: (1) growth hormone and insulin-like growth Factor-1 (IGF-1) which may be involved in controlling the development of NAFLD^50,51^, (2) T-cell protein tyrosine phosphatase (TCPTP) which leads to activation of both STAT-1 and STAT-3 signaling and may be important in the development of NASH HCC in patients with MS,^52^ and (3) Neuregulin 4, which suppresses NASH-HCC development by restraining the tumor-prone liver microenvironment.^53^ Also, the recent discovery that individuals with mutations in a gene (CIDEB) that codes for a structural protein in hepatic lipid droplets are protected from developing severe liver disease suggests that targeting this protein, or possibly biology associated with it, might be an avenue for preventing the development of severe liver disease including NAFLD/NASH.^49^

The primary limitation of this analysis is that it was a retrospective study with limited sample size and with incomplete data in some instances. This limits the ability to determine potentially statistically significant differences between the cohorts. The manual curation of data from chart review, however, did allow for high accuracy and granularity in data collection, and comparative analyses were restricted to variables with data available for the majority of patients. In addition, assessment of steatohepatitis was not reported on all pathology reports so the absence or presence of NAFLD/NASH could not be histologically confirmed in all cases by chart review. We addressed this limitation by collaborating with an attending pathologist who manually reviewed all cases for which tissue was available. We relied on the imperfect surrogate of the presence or absence of MS for the remaining cases and acknowledge that it is possible that some of these patients did not have NAFLD or NASH. Another limitation of this retrospective review is that surgical resection was not randomized and there may have been differences inherently in the cohorts selected for resection.

Continued efforts to better understand the biology of factors that determine the progression of NAFLD to NASH and ultimately HCC are critically important for the development of more effective therapeutic interventions as well as biomarkers of progression from NAFLD to NASH and ultimately HCC allowing for early intervention and treatment as appropriate.

In parallel, as new therapeutics and biomarkers are developed, lifestyle modifications—including dietary changes, sustained weight loss, and increased physical activity—remain the cornerstone of counsel to individual patients to decrease their risk of developing HCC.

## Supplementary Material

oyac251_suppl_Supplementary_Material

## Data Availability

The data underlying this article will be shared on reasonable request to the corresponding author.
