# Supplementary material for: Resection of NAFLD/NASH-related Hepatocellular Carcinoma (HCC): Clinical Features and Outcomes Compared with HCC Due to Other Etiologies
Source: Oncologist. 2023 Feb 10;28(4):341–50. doi: 10.1093/oncolo/oyac251 (PMC10078904; doi:10.1093/oncolo/oyac251)
Supplement: oyac251_suppl_Supplementary_Material [file oyac251_suppl_supplementary_material.docx]

**Supplemental Figure- S1**

**Recurrence Free Survival NAFLD/NASH VS VIRAL ONLY Cohorts**


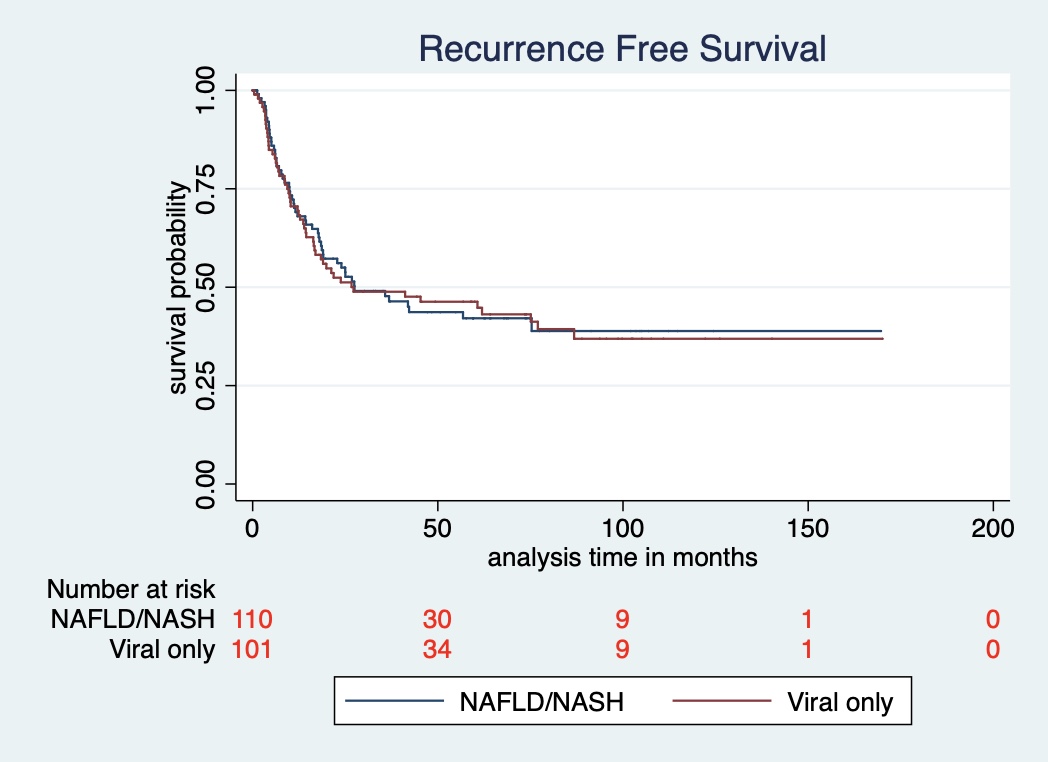


NAFLD/NASH: 27.5 months (95% CI 18.6-NA)

Viral only: 27.1 months,95% CI 16.5-86.8)

HR: 1.0, 95% (CI 0.7-1.4), p=0.9

**Supplemental Figure- S2**

**Overall Survival NAFLD/NASH vs Viral only cohorts**


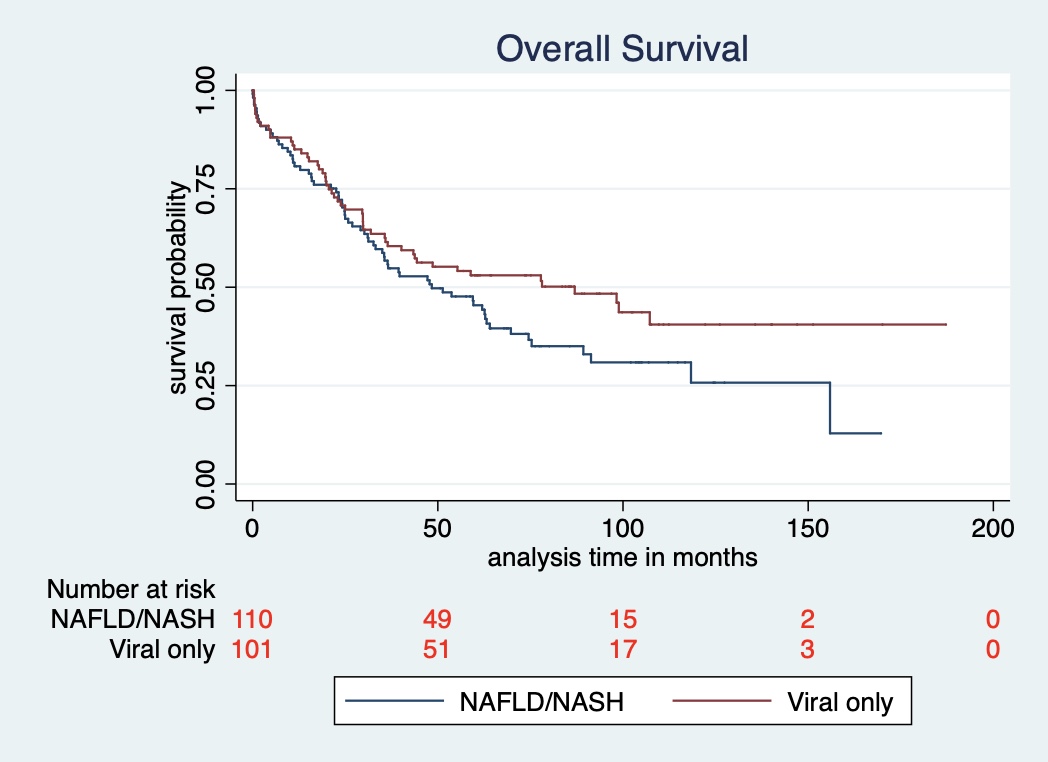


NAFLD/NASH:48.3 months (95% CI 32.6-64.0)

Viral only:86.9 months (95% CI 40.1-NA)

HR:0.73, 95% CI 0.51-1.0, p=0.095
